# Supplementary material for: Towards intense single-digit attosecond pulses with a 100-mJ-class mid-infrared sub-cycle laser
Source: Sci Adv. 2026 Oct 21;12(43):eaed5357. doi: 10.1126/sciadv.aed5357 (PMC13685965; doi:10.1126/sciadv.aed5357)
Supplement: Supplementary file 1 [file sciadv.aed5357_sm.pdf]

Science Advances
AAAS

Supplementary Materials for

Towards intense single-digit attosecond pulses with a 100-mJ-class mid-infrared sub-cycle laser

Kaito Nishimiya *et al*.

Corresponding author: Eiji J. Takahashi, ejtak@riken.jp

Sci. Adv. **12**, eaed5357 (2026)
DOI: 10.1126/sciadv.aed5357

This PDF file includes:

Supplementary Text
Fig. S1

Supplementary Text

Detail of the system layout

Figure S1 shows the detailed energy distribution and beam propagation in the HNC-DCOPA setup. In this system, it is important to propagate the beam with as large a beam size as possible to maintain the beam quality. If the collimated beam diameter is reduced below approximately 10 mm, the beam profile can degrade into a doughnut-like profile during propagation, leading to locally increased intensity on the amplification crystal.

In the first DCOPA stage, which operated at 1 kHz, the seed pulse was temporally stretched using a transmissive Treacy-type pulse stretcher and then propagated to the focusing lens of the DC-OPA through an image-relay optical system. In the first-stage amplifier, 30% of the pump energy was distributed to the BiBO crystal and 70% to the MgO:LN crystal. Both the pump and seed beams were focused into the nonlinear crystals for amplification, while an iris placed before the pump focusing lens was used to adjust the pump intensity in the crystals.

The second and third amplification stages were operated at a repetition rate of 10 Hz. The 10-Hz pump beam was S-polarized, with a beam diameter of approximately 6 cm and an energy of 760 mJ. Approximately 8% of this pump beam was first reflected by a window with an AR coating on one side and used for the second amplification stage, while the transmitted beam was used for the third amplification stage.

In the second DC-OPA stage, both the seed and pump beams were incident on the nonlinear crystals in a collimated geometry. The beam diameter on the crystals was approximately 4.8 mm. Before entering the second-stage DC-OPA, the reflected pump beam was reduced in size by a factor of 0.4 and then split by a beam splitter: 40% was used for the BiBO crystal and 60% for the MgO:LN crystal. Because the pump beam in the second-stage DC-OPA was particularly sensitive to beam-quality degradation, the beam was further reduced by a factor of 0.2 using a Keplerian telescope. The focal point of this telescope was placed in vacuum to avoid beam-profile degradation caused by ionization in air. After the second amplification stage, the amplified seed beam was focused onto a pinhole (250 μm diameter) using a silver concave mirror with a focal length of 25 cm, and the beam quality was improved by spatial filtering. The throughput of this spatial filtering was approximately 50%.

The seed beam diverging after the pinhole was recollimated using a silver concave mirror with a focal length of 75 cm and was then introduced into the third DC-OPA stage. The pump beam transmitted through the window was used for the third-stage amplifier. Before the beam splitter, the pump beam was reduced in size by a factor of 0.6 and then divided so that 30% was used for the BiBO crystal and 70% for the MgO:LN crystal. In the third DC-OPA stage, the pump beam diameter on the crystals was approximately 14 mm. After the third DC-OPA stage, the amplified beam was expanded by a factor of approximately 2.5 using a beam expander consisting of silver convex and concave mirrors and was subsequently compressed using a sapphire bulk compressor.

Optical path stabilization in SSG using the He-Ne laser

The feedback loop used a linearly polarized He–Ne laser (LGK7628, LASOS) with an output power of 5 mW. The relative optical path length was evaluated from the phase of the spatial interference pattern after Fourier transformation and a phase shift of 2π rad corresponds to an optical-path-length change of 632 nm. Although the beam splitter, beam combiner, and AOPDF in the SSG reflected or transmitted only part of the He–Ne beam, the remaining intensity of 30 μW was sufficient for stable phase detection. The interference signal was detected

using a CMOS camera (UI-1480SE-M-GL, IDS Imaging) with integration time of 50 ms. The phase signal was processed by a LabVIEW-based digital PI circuit, converted to an analog voltage using a D/A converter (NI 9263, National Instruments), and fed back to a piezo-driven translation stage (P-752.11C, PI) with a travel range of 20 μm. The feedback response time was approximately 1 s, and the interference fringe visibility was approximately 50%, which was sufficient for stable phase retrieval and feedback control.

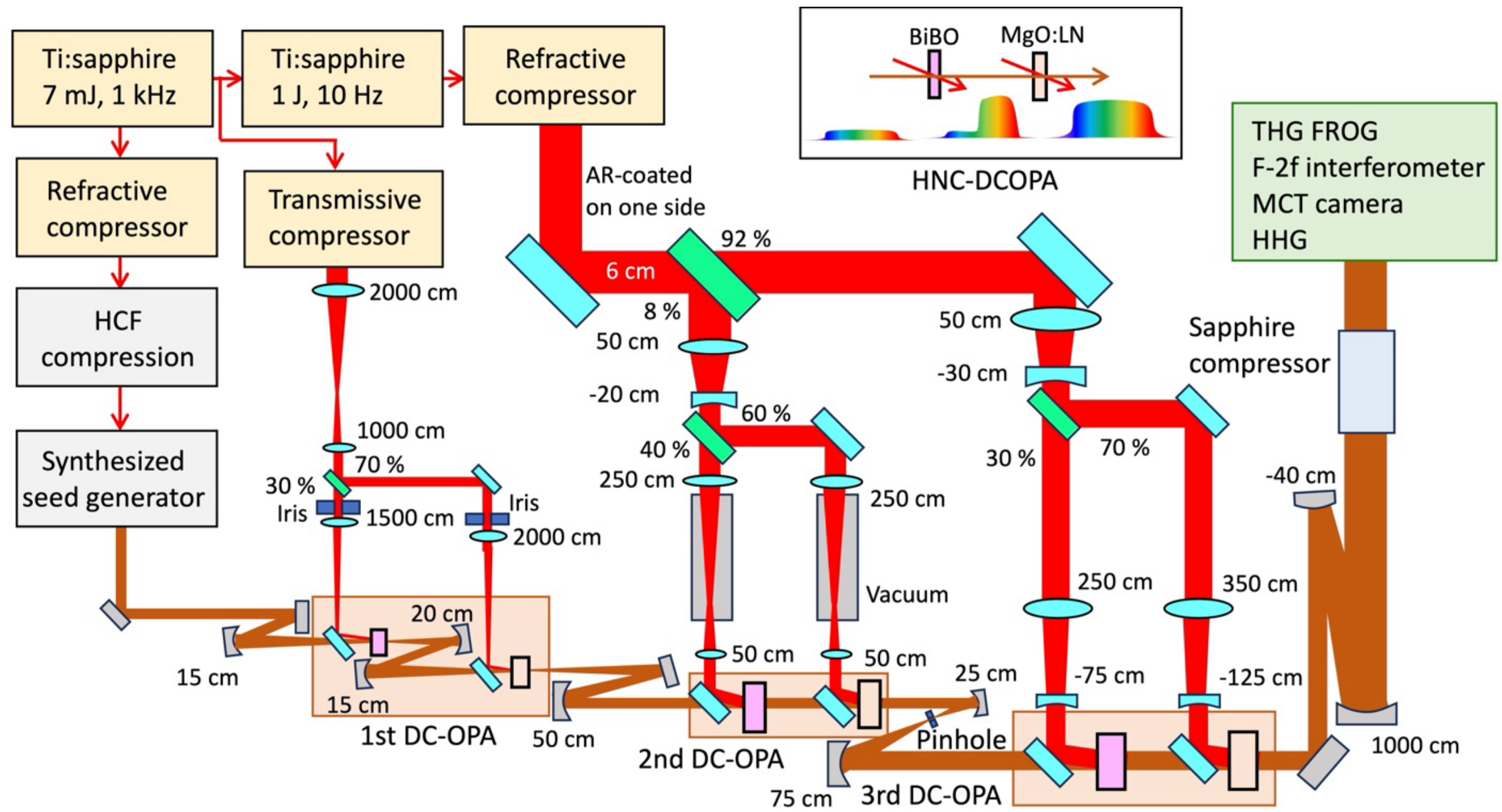

Fig. S1. Detailed schematic of beam propagation and energy distribution in the HNC-DCOPA system. The values next to the lenses and concave mirrors indicate their focal lengths, and the percentages shown at the beam splitters (green) indicate the energy fractions after splitting.
